# Matricryptins Network with Matricellular Receptors at the Surface of Endothelial and Tumor Cells

**DOI:** 10.3389/fphar.2016.00011

**Published:** 2016-02-04

**Authors:** Sylvie Ricard-Blum, Sylvain D. Vallet

**Affiliations:** University Claude Bernard Lyon 1, UMR 5246 Centre National de la Recherche Scientifique - University Lyon 1 - Institut National des Sciences Appliquées de Lyon - École Supérieure de Chimie Physique Électronique de LyonVilleurbanne, France

**Keywords:** matricryptins, endostatin, matricellular receptors, interaction networks, anticancer drugs

## Abstract

The extracellular matrix (ECM) is a source of bioactive fragments called matricryptins or matrikines resulting from the proteolytic cleavage of extracellular proteins (e.g., collagens, elastin, and laminins) and proteoglycans (e.g., perlecan). Matrix metalloproteinases (MMPs), cathepsins, and bone-morphogenetic protein-1 release fragments, which regulate physiopathological processes including tumor growth, metastasis, and angiogenesis, a pre-requisite for tumor growth. A number of matricryptins, and/or synthetic peptides derived from them, are currently investigated as potential anti-cancer drugs both *in vitro* and in animal models. Modifications aiming at improving their efficiency and their delivery to their target cells are studied. However, their use as drugs is not straightforward. The biological activities of these fragments are mediated by several receptor families. Several matricryptins may bind to the same matricellular receptor, and a single matricryptin may bind to two different receptors belonging or not to the same family such as integrins and growth factor receptors. Furthermore, some matricryptins interact with each other, integrins and growth factor receptors crosstalk and a signaling pathway may be regulated by several matricryptins. This forms an intricate 3D interaction network at the surface of tumor and endothelial cells, which is tightly associated with other cell-surface associated molecules such as heparan sulfate, caveolin, and nucleolin. Deciphering the molecular mechanisms underlying the behavior of this network is required in order to optimize the development of matricryptins as anti-cancer agents.

## Introduction

Matricryptins are biologically active fragments released from extracellular matrix (ECM) proteins and glycosaminoglycans by proteases (Davis et al., 2000). We have extended the definition of matricryptins to the ectodomains of membrane collagens and membrane proteoglycans, which are released in the ECM by sheddases, and to fragments of ECM-associated enzymes such as lysyl oxidase, which initiates the covalent cross-linking of collagens and elastin, and matrix metalloproteinases (MMPs), which contribute to ECM remodeling (Ricard-Blum and Salza, 2014; Ricard-Blum and Vallet, 2015). The molecular functions of matricryptins and the biological processes they regulate have been reviewed with a focus on collagen and proteoglycan matricryptins (Ricard-Blum and Ballut, 2011), on matricryptins regulating tissue repair (Ricard-Blum and Salza, 2014), angiogenesis (Sund et al., 2010; Boosani and Sudhakar, 2011; Gunda and Sudhakar, 2013), cancer (Monboisse et al., 2014), and on the proteases releasing matricryptins (Ricard-Blum and Vallet, 2015).

Synthetic peptides and/or domains derived from matricryptin sequences and recapitulating their biological roles are also able to regulate angiogenesis and/or cancer in various tumor cells and cancer models (Rosca et al., 2011). They include sequences of tumstatin (He et al., 2010; Han et al., 2012; Wang et al., 2015a), laminins (Kikkawa et al., 2013), endostatin (Morbidelli et al., 2003), endorepellin (Willis et al., 2013), and the hemopexin domain of MMP-9 (Ugarte-Berzal et al., 2012, 2014). Neither these peptides nor the ectodomains of membrane collagens and syndecans are described here due to space limitation. We focus on the major matricryptins, which control cancer, metastasis, and angiogenesis, a pre-requisite for tumor growth and a therapeutic target (Folkman, 1971; Welti et al., 2013; Huang et al., 2015), and on their receptors.

## Regulation of angiogenesis, tumor growth and metastasis by matricryptins

Matricryptins regulate wound healing, fibrosis, inflammation, angiogenesis, and cancer and are involved in infectious and neurodegenerative diseases (Ricard-Blum and Ballut, 2011; Ricard-Blum and Salza, 2014; Ricard-Blum and Vallet, 2015). Most of the matricryptins regulating angiogenesis and tumor growth are derived from collagens IV and XVIII (Monboisse et al., 2014; Walia et al., 2015), elastin (Robinet et al., 2005; Pocza et al., 2008; Heinz et al., 2010), fibronectin (Ambesi et al., 2005), laminins (Tran et al., 2008), osteopontin (Bayless and Davis, 2001; Lund et al., 2009; Yamaguchi et al., 2012), MMPs (Bello et al., 2001; Ezhilarasan et al., 2009), proteoglycans (Goyal et al., 2011), and hyaluronan (Cyphert et al., 2015; Table 1). They are released from the ECM by a variety of proteinases (matrixins, adamalysins, tolloids, cathepsins, thrombin, and plasmin; Ricard-Blum and Vallet, 2015; Wells et al., 2015).

**Table 1:** **Matricryptins, receptors, and signaling pathways regulated by matricryptins in endothelial and tumor cells**.

**Receptors**	**Matricryptins**	**Signaling pathways**	**Cells**	**References**
**INTEGRINS**				
α1β1	Arresten *(α1 chain of collagen IV)*	Inhibition of FAK/c-Raf/MEK1/2/ERK1/2/p38 MAPK pathway; Inhibition of hypoxia-induced expression of HIF 1α and VEGF	ECs	Sudhakar et al., 2005
			HSC-3 human tongue squamous carcinoma cells	Aikio et al., 2012
α2β1	Endorepellin *(C-terminus of perlecan)*	Activation of SHP-1	ECs	Nyström et al., 2009
		Activation of the tyrosine phosphatase SHP-1; Dephosphorylation of VEGFR2; Down-regulation of VEGFA	ECs	Goyal et al., 2011
		Down-regulation of VEGFR2	ECs	Poluzzi et al., 2014
	Procollagen I C-propeptide		HT1080 human fibrosarcoma cells	Weston et al., 1994
α3β1	Tumstatin *(α3 chain of collagen IV)*	Integrin α3β1 is a trans-dominant inhibitor of integrin αv	ECs	Borza et al., 2006
	Canstatin *(α2 chain of collagen IV)*		ECs	Petitclerc et al., 2000
α4β1	N-terminal osteopontin fragment		HL-60 human promyelocytic leukemia cells	Bayless and Davis, 2001
	PEX domain of MMP-9		Human chronic lymphocytic leukemia B cells	Ugarte-Berzal et al., 2012
α4β7	N-terminal osteopontin fragment		RPMI 8866 human lymphoblastoid cell line	Green et al., 2001
α5β1	Endostatin *(α1 chain of collagen XVIII)* K_D_ = 975 and 451 nM, 2 binding sites, soluble endostatin, immobilized full-length integrin; (Faye et al., 2009b)	Inhibition of FAK/c-Raf/MEK1/2/p38/ERK1 MAPK pathway	ECs	Sudhakar et al., 2003
		Induction of phosphatase-dependent activation of caveolin-associated Src family kinases	ECs	Wickström et al., 2002
		Induction of recruitment of α5β1 integrin into the raft fraction via a heparan sulfate proteoglycan-dependent mechanism.	ECs	Wickström et al., 2003
		Induction of Src-dependent activation of p190RhoGAP with concomitant decrease in RhoA activity and disassembly of actin stress fibers and focal adhesions		
			Hemangioendothelioma-derived cells	Guo et al., 2015
	N-terminal osteopontin fragment		Human colorectal adenocarcinoma (SW480 cells)	Yokosaki et al., 2005
α6β1	Tumstatin *(α3 chain of collagen IV)*		ECs	Maeshima et al., 2000
α9β1	N-terminal osteopontin fragment		Human colorectal adenocarcinoma (SW480 cells)	Yokosaki et al., 2005
αvβ3	Endostatin *(α1 chain of collagen XVIII)* K_D_ = 1.2 μM and 501 nM, 2 binding sites, soluble endostatin, immobilized full-length integrin; (Faye et al., 2009b)		ECs	Rehn et al., 2001
	Canstatin *(α2 chain of collagen IV)*	Induction of two apoptotic pathways through the activation of caspase-8 and caspase-9	ECs	Magnon et al., 2005
		Induction of caspase 9-dependent apoptotic pathway	Human breast adenocarcinoma cells (MDA-MB-231)	Magnon et al., 2005
			ECs	Petitclerc et al., 2000
	Tumstatin *(α3 chain of collagen IV)*	Inhibition of Cap-dependent translation (protein synthesis) mediated by FAK/PI3K/Akt/mTOR/4E-BP1 pathway	ECs	Maeshima et al., 2000; Sudhakar et al., 2003
			ECs	Petitclerc et al., 2000
		Inhibition of the activation of FAK, PI3K, protein kinase B (PKB/Akt), and mTOR	ECs	Maeshima et al., 2002
		It prevents the dissociation of eukaryotic initiation factor 4E protein from 4E-binding protein 1		
		Stimulation of FAK and PI3K phosphorylation	Human metastatic melanoma cell line (HT-144)	Pasco et al., 2000
		Inhibition of the growth of tumors dependent on Akt/mTOR activation (functional PTEN required)	Human glioma cells	Kawaguchi et al., 2006
	Tetrastatin *(α4 chain of collagen IV)* K_D_ = 148 nM (2-state model, soluble tetrastatin, immobilized full-length integrin)		Human melanoma cells (UACC-903)	Brassart-Pasco et al., 2012
	NC1 domain of α6 chain of collagen IV		ECs	Petitclerc et al., 2000
	Procollagen II N-propeptide		Human chondrosarcoma cell line (hCh-1)	Wang et al., 2010
	PEX domain of MMP-2		ECs	Brooks et al., 1998
	N-terminal osteopontin fragment		Human colorectal adenocarcinoma (SW480 cells)	Yokosaki et al., 2005
	*VGAPG, VGAP (elastin peptides)*		Human melanoma cell lines (WM35 and HT168-M1)	Pocza et al., 2008
αvβ5	Endostatin *(α1 chain of collagen XVIII)*		ECs	Rehn et al., 2001
	Canstatin *(α2 chain of collagen IV)*	Induction of two apoptotic pathways through the activation of caspase-8 and caspase-9	ECs	Magnon et al., 2005
		Induction of caspase 9-dependent apoptotic pathway	Human breast adenocarcinoma cells (MDA-MB-231)	Magnon et al., 2005
			ECs	Petitclerc et al., 2000
	Tumstatin *(α3 chain of collagen IV)*		ECs	Pedchenko et al., 2004
	Procollagen II N-propeptide		Human chondrosarcoma cell line (hCh-1)	Wang et al., 2010
	N-terminal osteopontin fragment		Human colorectal adenocarcinoma (SW480 cells)	Yokosaki et al., 2005
αvβ6	N-terminal osteopontin fragment		Human colorectal adenocarcinoma (SW480 cells)	Yokosaki et al., 2005
**GROWTH FACTOR RECEPTORS**				
VEGFR1	Endostatin *(α1 chain of collagen XVIII)*		ECs	Kim et al., 2002
	Endorepellin *(C-terminus of perlecan)* K_D_ = 1 nM (soluble endorepellin, immobilized ectodomain of VEGFR1)		ECs	Goyal et al., 2011
VEGFR2	Endostatin *(α1 chain of collagen XVIII)*	Inhibition of VEGF-induced tyrosine phosphorylation of VEGFR2 and activation of ERK, p38 MAPK, and p125FAK	ECs	Kim et al., 2002
	Endorepellin *(C-terminus of perlecan)*	Attenuation of VEGFA-evoked activation of VEGFR2 at Tyr^1175^	ECs	Goyal et al., 2011
		K_D_ = 0.9 nM (soluble endorepellin, immobilized ectodomain of VEGFR2)		
		Attenuation of both the PI3K/PDK1/Akt/mTOR and the PKC/JNK/AP1 pathways	ECs	Goyal et al., 2012
		Induction of the formation of the Peg3-Vps34-Beclin 1 autophagic complexes via inhibition of the PI3K/Akt/mTOR pathway	ECs	Poluzzi et al., 2014
		Induction of autophagy through a VEGFR2 dependent but α2β1 integrin-independent pathway		
EGFR	Laminin-332 EGF-like (domain III) of the γ2 chain	Stimulation of EGFR phosphorylation; Induction of ERK phosphorylation	Human breast adenocarcinoma cells (MDA-MB-231)	Schenk et al., 2003
**CHEMOKINE RECEPTORS**				
CXCR2	Proline-glycine-proline *(collagen matrikine)*	Activation of Rac1, increase in phosphorylation of ERK, PAK and VE-cadherin	ECs	Hahn et al., 2015
**HEPARAN SULFATE PROTEOGLYCANS**				
Glypican-1	Endostatin *(α1 chain of collagen XVIII)*		ECs	Karumanchi et al., 2001
Glypican-4	Endostatin *(α1 chain of collagen XVIII)*		ECs	Karumanchi et al., 2001
Syndecan-1	LG45 domain of the α3 chain of laminin-332		HT1080 human fibrosarcoma cells	Carulli et al., 2012
Syndecan-4	LG45 domain of the α3 chain of laminin-332		HT1080 human fibrosarcoma cells	Carulli et al., 2012
**ELASTIN RECEPTOR COMPLEX**				
Elastin receptor complex	Elastin peptides (xGxxPG sequences)	67 kDa elastin binding protein (an alternatively spliced form of β-galactosidase)	ECs	Robinet et al., 2005
			Human melanoma cell lines (WM35 and HT168-M1)	Pocza et al., 2008
**GALECTIN-3 RECEPTOR**				
Galectin-3 receptor	VGVAPG and VAPG *(elastin peptides)*		Human melanoma cell lines (WM35 and HT168-M1)	Pocza et al., 2008
**LACTOSE-INSENSITIVE RECEPTOR**				
Lactose-insensitive receptor	VGVAPG *(elastin peptide)*		M27 subline of murine Lewis lung carcinoma	Blood and Zetter, 1993
	AGVPGLGVG and AGVPGFGAG *(elastin peptides)*		Human lung carcinoma cells	Toupance et al., 2012
**CD44, RHAMM AND TLR4**				
CD44	Hyaluronan oligosaccharides (3–10 disaccharides)	PKC-α phosphorylation of γ-adducin, a membrane cytoskeletal and actin-binding protein, Activation of ERK1/2	ECs	Matou-Nasri et al., 2009
		Stimulation of ERK1/2 signaling Inhibition of CD44 clustering (3–10 disaccharides)	Human breast cancer cells (BT-159, ductal carcinoma)	Yang et al., 2012
	N-terminal osteopontin fragment (Leu^1^-Gly^127^)	CD44-mediated OPN binding requires β1 integrin	Rat BDX2 fibrosarcoma cells	Katagiri et al., 1999
	C-terminal osteopontin fragment (Leu^132^-Asn^278^)	CD44-mediated OPN binding requires β1 integrin	Rat BDX2 fibrosarcoma cells	Katagiri et al., 1999
	Osteopontin fragment (5 kDa, residues 167–210)		Human hepatocellular carcinoma cells	Takafuji et al., 2007
	PEX domain of MMP-9		Human chronic lymphocytic leukemia cells	Ugarte-Berzal et al., 2014
LYVE-1	Hyaluronan oligosaccharides (3–10 disaccharides)	Increased tyrosine phosphorylation of protein kinase Cα/βII and ERK1/2	ECs	Wu et al., 2014
TLR4	Hyaluronan oligosaccharides (4, 6, 8-mer HA fragments)		ECs	Taylor et al., 2004
RHAMM	Hyaluronan oligosaccharides (2–10 disaccharides)	Activation of ERK1/2	ECs	Gao et al., 2008
	Hyaluronan oligosaccharides (3–10 disaccharides)	Activation of ERK1/2 Up-regulation of cdk1/Cdc2		Matou-Nasri et al., 2009
**CELL SURFACE ASSOCIATED PROTEIN**				
Nucleolin	Endostatin *(α1 chain of collagen XVIII)* K_D_ = 13 nM; (Shi et al., 2007)		Hemangioendothelioma-derived cells	Guo et al., 2015

*Receptors identified in other cell types and the associated signaling pathways are mentioned in the text. 4E-BP1, eukaryotic translation initiation factor 4E-binding protein 1; AP1, activation protein 1; Cdk1/Cdc2, cyclin-dependent kinase-1; CXCR2, CXC chemokine receptor 2; CXCL1, Chemokine (C-X-C motif) ligand 1; EC, endothelial cell; EGFR, epidermal growth factor receptor; ERK, extracellular signal-regulated kinase; FAK, focal adhesion kinase; HIF, hypoxia-inducible factor; JNK, c-Jun N-terminal kinases; LG, laminin G domain-like; LYVE-1, Lymphatic vessel endothelial hyaluronan receptor 1; MAPK, mitogen-activated protein kinase; MEK, mitogen-activated protein kinase kinase; MMP, matrix metalloproteinase; PAK, p21-activated kinase; PDK, phosphoinositide-dependent kinase; PI3K, phosphatidylinositol 3-kinase; PKB, protein kinase B; PKC, protein kinase C; PTEN, phosphatase and tensin homolog; RHAMM, receptor for HA-mediated motility; SHP-1, Src homology-2 protein phosphatase-1; TLR4, Toll-like receptor 4; VE-cadherin, vascular endothelial cadherin; VEGFA, vascular endothelial growth factor A; VEGFR, Vascular endothelial growth factor receptor*.

Matricryptins regulating angiogenesis and tumor growth target endothelial cells and/or tumor cells (Robinet et al., 2005; Tran et al., 2008; Sund et al., 2010; Boosani and Sudhakar, 2011; Ricard-Blum and Ballut, 2011; Toupance et al., 2012; Kikkawa et al., 2013; Monboisse et al., 2014; Ricard-Blum and Salza, 2014; Monslow et al., 2015; Nikitovic et al., 2015; Ricard-Blum and Vallet, 2015; Walia et al., 2015). Several matricryptins inhibit the proliferation and the migration of endothelial cells, block cell cycle at G1 as shown for anastellin (Ambesi et al., 2005) and endostatin (Hanai et al., 2002) and induce apoptosis. Arresten, derived from the C-terminus of the α1 chain of collagen IV, activates FasL mediated apoptosis for example (Verma et al., 2013). Endostatin and endorepellin, a matricryptin of perlecan, induce autophagy of endothelial cells, the autophagic activity of endorepellin being mediated by a VEGFR2-dependent pathway (Nguyen et al., 2009; Poluzzi et al., 2014). A modified endostatin (Endostar) induces autophagy in hepatoma cells (Wu et al., 2008). Matricryptins normalize tumor vasculature, which improves the delivery of cytotoxic drugs to the tumor and hence the response to anti-cancer treatments (Jain, 2005). Endostatin contributes to the normalization of tumor vasculature in a lung cancer model (Ning et al., 2012), and in esophageal squamous cell carcinoma, where it enhances the effect of radiotherapy and reduces hypoxia (Zhu et al., 2015), possibly by a crosstalk between cancer and endothelial cells mediated by the Hypoxia-Inducible Factor and VEGF expression.

Matricryptins derived from collagens IV and XVIII target tumoral cells. Arresten inhibits migration and invasion of squamous cell carcinoma and induces their death (Aikio et al., 2012). Endostatin inhibits the proliferation of some cancer cells (e.g., the HT29 human colorectal adenocarcinoma cell line) but not of others (e.g., the MDA-MB-231 human mammary adenocarcinoma cell line) (Ricard-Blum et al., 2004). Matricryptins enhance the sensitivity of tumor cells to a cytotoxic drug and even reverse in part their resistance to this drug. A tumstatin peptide increases the sensitivity of non-small cell lung carcinoma cells to cisplatin (Wang et al., 2015c) and Endostar enhances the sensitivity to radiation of nasopharyngeal carcinoma and lung adenocarcinoma xenografts in mice (Wen et al., 2009).

Matricryptins regulate angiogenesis, tumor growth, and metastasis by various molecular mechanisms. The anti-angiogenic activities of tumstatin and endostatin contribute to tumor suppression by p53 *via* the upregulation of the α(II) collagen prolylyl hydroxylase (Folkman, 2006; Teodoro et al., 2006). Endostatin inhibits proliferation and migration of glioblastoma cells by inhibiting T-type Ca^2+^ channels (Zhang et al., 2012), and its ATPase activity contributes to its anti-angiogenic and antitumor properties (Wang et al., 2015b). This matricryptin inhibits hemangioendothelioma by downregulating chemokine (C-X-C motif) ligand 1 *via* the inactivation of NF–κB (Guo et al., 2015).

## Receptors and co-receptors of matricryptins

**Figure 1:**
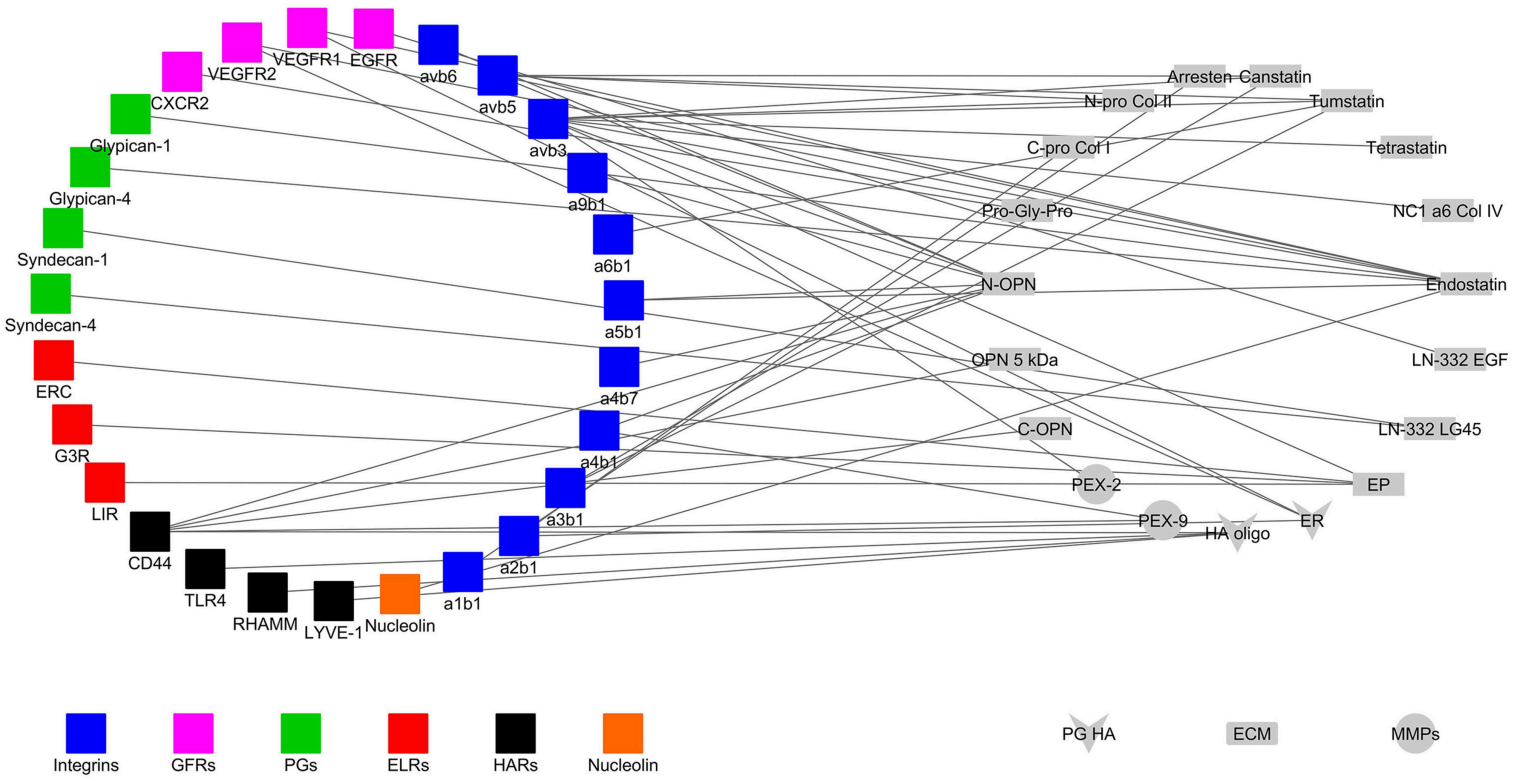
**Interaction network of matricryptins (right) and their receptors (left) expressed at the surface of endothelial and cancer cells**. ab, alpha and beta integrin subunits; C-Pro Col, C-propeptide of procollagen; CXCR, chemokine CXC receptor; ECM, extracellular matrix; EGF, epidermal growth factor; EGFR, epidermal growth factor receptor; EP, elastin peptide; ER, endorepellin; ERC, elastin receptor complex; ELR, elastin receptor; ES, endostatin; G3R, galectin-3 receptor; GFR, growth factor receptor; HA oligo, hyaluronan oligosaccharide; HAR, hyaluronan receptor; LN LG45, laminin domain LG45; LIR, lactose insensitive receptor; LYVE-1, lymphatic vessel endothelial hyaluronan receptor 1; N-Pro Col, N-propeptide of procollagen; MMP, matrix metalloproteinase; NC1, non-collagenous domain; OPN, osteopontin; PEX, hemopexin domain; PG, proteoglycan; RHAMM, receptor for hyaluronic acid-mediated motility; TLR4, toll-like receptor; VEGFR, vascular endothelial growth factor receptor.

Matricryptins regulating angiogenesis, tumor growth and metastasis bind to several receptors, and co-receptors (Figure 1, Faye et al., 2009a) to modulate signaling pathways and fulfill their biological functions (Table 1). The other ligands of the receptors (e.g., ECM proteins, proteoglycans, growth factors, and chemokines) are not represented in Figure 1 for the sake of clarity. Pathways regulated by matricryptins in endothelial or tumor cells *via* unidentified receptors and/or in other cell types are mentioned below but are not listed in Table 1.

### Integrins

There are 24 integrins comprised of an α subunit and a β subunit (Barczyk et al., 2010). They lack intrinsic kinase activity and are the major adhesion receptors of the ECM, controlling ECM assembly, cell-matrix interactions, cell migration, and tumor growth (Missan and DiPersio, 2012; Xiong et al., 2013). A number of matricryptins bind to integrins at the surface of tumor and/or endothelial cells (Table 1). Matricryptins also interact with purified integrins (e.g., αvβ5 integrin for endostatin; Rehn et al., 2001; Faye et al., 2009b), or on other cell types. The αvβ3 integrin is the main receptor targeted by matricryptins (Figure 1).

Anastellin decreases the activation state of α5β1 integrin on endothelial cells (Ambesi and McKeown-Longo, 2014). Arresten interacts with α3β1/αvβ3 and α1β1/α2β1 integrins at the surface of HPV-16-immortalized proximal tubular epithelial cells and mesangial cells respectively, whereas tumstatin binds to immortalized glomerular epithelial cells through α3β1 and α2β1 integrins (Aggeli et al., 2009). The above integrins are also involved in the effects of matricryptins on other cell types. Endostatin, generated by cerebellar Purkinje cells, contributes to the organization of climbing fiber terminals in these neurons by binding and signaling through α3β1 integrin (Su et al., 2012). The adhesion of smooth muscle cells to anastellin is mediated by both β1 integrins and cell surface heparan sulfate proteoglycans, which triggers ERK1/2 activation in these cells (Mercurius and Morla, 2001) and the induction of osteoclast apoptosis by the N-propeptide of procollagen II is mediated by αv or β3 integrin subunits (Hayashi et al., 2011).

### Growth factor and chemokine receptors

Growth factor receptors belong to the tyrosine kinase receptor family. They regulate cell proliferation, differentiation, cell cycle, survival and apoptosis and play a role in cancer (McDonell et al., 2015). VEGR receptors 1–3 (Roskoski, 2008; Grünewald et al., 2010; Simons, 2012) and EGF receptor (Lemmon et al., 2014) interact with matricryptins (Table 1). Two endostatin peptides bind to VEGFR3 (Han et al., 2012, 2015) and EGF-like repeats of tenascin C interact with EGFR, inducing phosphorylation of the receptor and of ERK MAP kinases in NR6 mouse fibroblasts (Swindle et al., 2001). Endorepellin simultaneously engages VEGFR2 and α2β1 integrin, both expressed by endothelial cells, and regulate angiogenesis and autophagy through a dual receptor antagonism (Goyal et al., 2011; Poluzzi et al., 2014). Anastellin inhibits lysophospholipid (Ambesi and McKeown-Longo, 2009) and VEGF165-dependent signaling in endothelial cells by preventing the formation of the complex containing VEGFR2 and neuropilin-1 at the surface of endothelial cells (Ambesi and McKeown-Longo, 2014). One matricryptin of collagen I interacts with a member of the chemokine receptor family, the CXC chemokine receptor 2 (Stadtmann and Zarbock, 2012; Veenstra and Ransohoff, 2012).

### Cell surface proteoglycans

Proteoglycans are divided into intracellular, pericellular, extracellular, and cell-surface families (Iozzo and Schaefer, 2015). Syndecans are transmembrane heparan sulfate proteoglycans (Couchman et al., 2015), which play a role in cell adhesion, migration, receptor trafficking, growth factor interactions, angiogenesis (De Rossi and Whiteford, 2014) and cancer (Barbouri et al., 2014). They are enzymatically shed from the cell surface and compete with their membrane forms for ligand binding (Manon-Jensen et al., 2010). They act in synergy with integrins to control cell adhesion and other biological processes (Morgan et al., 2007; Roper et al., 2012; Humphries et al., 2015), and the binding of heparan sulfate chains to integrin α5β1 promotes cell adhesion and spreading (Faye et al., 2009b). Syndecans act as co-receptors of VEGF and control tumor progression in association with integrins (Grünewald et al., 2010; Soares et al., 2015). Glypicans, membrane-associated proteoglycans with a glycosylphosphatidyl anchor, regulate Wnt, Hedgehog, fibroblast growth factor, and bone morphogenetic protein signaling (Filmus et al., 2008; Iozzo and Schaefer, 2015). One matricryptin, endostatin, binds to glypicans *via* their heparan sulfate chains (Karumanchi et al., 2001).

### Elastin receptors

The Elastin Receptor Complex (ERC) is composed of two membrane associated proteins (the protective protein/cathepsin A and neuraminidase-1) and of the elastin-binding protein, an inactive spliced variant of lysosomal β-galactosidase (Blanchevoye et al., 2013). Elastin peptides activate extracellular signal-regulated kinase 1/2 *via* a Ras-independent mechanism in fibroblasts (Duca et al., 2005), the enzymatic activity of neuraminidase-1 being responsible for signal transduction (Duca et al., 2007). Another, still unidentified, receptor of elastin peptides exists at the surface of macrophages (Maeda et al., 2007). Furthermore, elastin peptides regulate tumor cell migration and invasion through an Hsp90-dependent mechanism (Donet et al., 2014).

### CD44, receptor for HA-mediated motility (RHAMM) and toll-like receptors (TLRs)

Hyaluronan, a non-sulfated glycosaminoglycan, has two major receptors, CD44 and RHAMM, which mediate its roles in inflammation and cancer (Toole, 2009; Misra et al., 2015; Nikitovic et al., 2015). The binding to, and activation of, receptors depend on the size of HA, its oligosaccharides stimulating angiogenesis (Gao et al., 2008). CD44, which has several isoforms regulates cell proliferation, adhesion and migration, and is involved in tumorigenesis (Jordan et al., 2015). A sequence in the hemopexin domain of MMP-9 (PEX9) impairs tumor cell adhesion to PEX9/MMP9 through interaction with CD44 (Ugarte-Berzal et al., 2014). RHAMM has splicing variants and is located inside the cell and on the cell surface, where it is anchored *via* a glycosylphosphatidylinositol (Tolg et al., 2014; Misra et al., 2015). Toll-like receptors are pattern recognition receptors involved in innate immunity (Rakoff-Nahoum and Medzhitov, 2009). Low-molecular weight hyaluronan induces the formation of a complex containing CD44, TLR2/TLR4, the actin filament-associated protein AFAP-110, and a myeloid differentiation factor MyD88, which triggers cytoskeleton activation and results in tumor invasion (Bourguignon et al., 2011).

### Other membrane and cell surface-associated proteins

Matricryptins form complexes with membrane or membrane-associated proteins. Caveolin- participates in the formation of membrane caveolae, which are platforms for signal transduction (Fridolfsson et al., 2014) and forms a complex with α5β1 integrin and endostatin in lipid rafts at the endothelial cell surface (Wickström et al., 2002) (Table 1). Nucleolin, a multifunctional protein, localized inside the cell and at the cell surface (Berger et al., 2015), binds to endostatin and triggers its internalization in endothelial cells in association with the urokinase plasminogen activator receptor and the α5β1integrin (Shi et al., 2007; Song et al., 2012).

## Matricryptins as potential drugs

Matricryptins are potential anti-cancer drugs, either alone, or in combination with other treatments, but their use in pre-clinical and clinical studies remains challenging. Indeed matricryptins may display opposite activities depending on the context. The anti-tumoral effect of endostatin is enhanced by silencing of the proteoglycan versican, which decreases the inflammatory and immunosuppressive changes triggered by anti-angiogenic therapy (Wang et al., 2015d). However, endostatin may induce the proliferation of carcinoma cells, whereas its effect on cancer invasion is modulated by the tumor microenvironment (Alahuhta et al., 2015). Endorepellin stimulates angiogenesis in a stroke model by increasing VEGF levels, and exerts a neuroprotective effect in this model *via* α5β1 integrin and VEGFR2 (Lee et al., 2011). In addition, endostatin exhibits a biphasic response curve both for its anti-angiogenic and anti-tumoral properties (Celik et al., 2005; Javaherian et al., 2011), which requires to test a large range of concentrations to determine the optimal dose for treatment. Another challenge is that matricryptins may themselves contain cryptic sequences displaying opposite activities as reported for the anti-angiogenic matricryptin endostatin, which contains an embedded pro-angiogenic sequence (Morbidelli et al., 2003). Different matricryptins regulate the same biological process in an opposite way as reported for the regulation of the angiogenic signaling in choroidal endothelial cells by hexastatin and elastokines (Gunda and Sudhakar, 2013), or distinct steps of a biological process as described for anastellin and endostatin (Neskey et al., 2008).

Matricryptins can be modified to extend the half-life, and their efficacy (Xu et al., 2007; Zheng, 2009; Ricard-Blum and Ballut, 2011; Ricard-Blum and Salza, 2014). Most of the examples detailed below concern endostatin, which is extensively studied and has been approved for the treatment of non-small-cell lung cancer in China (Biaoxue et al., 2012) under a recombinant form, Endostar, which contains an extra metal chelating sequence (MGGSHHHHH) at the N-terminus enhancing its solubility and stability (Jiang et al., 2009). PEGylation (Nie et al., 2006; Tong et al., 2010; Tan et al., 2012; Guo et al., 2014), and the fusion of endostatin to low molecular weight heparin or to the Fc region of an IgG enhance its half-life and its anti-angiogenic, or anti-tumoral activities (Lee et al., 2008; Jing et al., 2011; Ning et al., 2012; Tan et al., 2012; Li et al., 2015b).

Tumors may escape from anti-tumoral and anti-angiogenic matricryptins by upregulating factors, which stimulate angiogenesis (Fernando et al., 2008). The combination of matricryptins with inhibitors of pro-angiogenic pathways, chemotherapy, or radiotherapy enhance their therapeutic efficacy. Tumstatin has been fused to another endogenous inhibitor of angiogenesis, vasostatin (Gu et al., 2014) and to tumor necrosis factor α, which has anti-tumoral and anti-angiogenic properties, which results in a more effective fusion protein than tumstatin alone (Luo et al., 2006). Endostatin has been fused to the proapoptotic domain (BH3) of the BAX protein (Chura-Chambi et al., 2014), to tumor necrosis factor-related apoptosis-inducing ligand (Zheng et al., 2013) and one of its anti-angiogenic sequences to an heptapeptide inhibitor of MMPs (Qiu et al., 2013). Endostatin has also been fused to protein sequences targeting it to tumors and/or tumor vasculature such as humanized antibodies against tyrosine kinase-type receptor HER2 (Shin et al., 2011) or against tumor-associated glycoprotein 72 highly expressed in human tumor tissues (Lee et al., 2015), the RGD integrin-binding sequence (Jing et al., 2011), and a liver-targeting peptide (circumsporozoite protein CSP I-plus (Ma et al., 2014; Bao et al., 2015).

Several approaches improve the delivery of matricryptins to tumors and endothelial cells (Xu et al., 2007; Ricard-Blum and Ballut, 2011) such as conditionally replicating oncolytic adenoviral vector for arresten (Li et al., 2015a), naked plasmid electrotransfer in muscle for tumstatin overexpression (Thevenard et al., 2013), and the nonpathogenic and anaerobic bacterium, *Bifidobacterium longum*, which proliferates in the hypoxic zones within tumors for tumstatin expression (Wei et al., 2015). Endostatin has been delivered in polylactic acid nanoparticles (Hu and Zhang, 2010), in gold nanoshells, which are very efficient on lung cancer cells when associated with near-infrared thermal therapy (Luo et al., 2015) and into microbubbles in combination with ultrasonic radiation in a cancer model (Zhang et al., 2014). Dendrimers mimicking the surface structure of endostatin and loaded with anticancer drug, result in both angiogenesis inhibition by endostatin and death of cancer cells by the anticancer drug (Jain and Jain, 2014).

Clinical trials of endostatin mostly focus on solid tumors in association with cytotoxic drugs (https://clinicaltrials.gov/). These trials include phase I (Lin et al., 2007; Chen et al., 2014), II (Lu et al., 2015), and III trials (Wang et al., 2005). Although endostatin did not improve overall survival, progression-free survival, and objective response rate when combined with etoposide and carboplatin in patients with extensive-stage small-cell lung cancer phase II trial (Lu et al., 2015), it significantly improves the response rate and median time to tumor progression when combined with vinorelbine and cisplatin in advanced non-small cell lung cancer patients compared to chemotherapy alone (Wang et al., 2005). Promising results have been obtained with endostatin associated with paclitaxel and 5-fluorouracile in patients with refractory malignant ascites secondary to ovarian cancer (Zhao et al., 2014).

## Concluding remarks

Several matricryptins such as the propeptide of lysyl oxidase, which is a tumor suppressor (Min et al., 2007; Ozdener et al., 2015) and the netrin-like domain of procollagen C-proteinase enhancer-1, a new anti-angiogenic matricryptin (Salza et al., 2014), warrant further investigation in angiogenesis, and tumor models to decipher their mechanisms of action at the molecular and cellular levels. Matricryptins are useful for treating fibroproliferative disorders (Yamaguchi et al., 2012; Wan et al., 2013) and *fundus oculi* angiogenesis diseases (Zhang et al., 2015). The finding that a peptide derived from endostatin can be delivered orally *in vivo* and exerts anti-fibrotic activity (Nishimoto et al., 2015) paves the way for the development of new matricryptin drugs with oral bioavailability, which is a preferred administration route for long-term treatment. Matricryptins are also used as biomarkers in serum and in cerebrospinal fluid (Ricard-Blum and Vallet, 2015; Salza et al., 2015) and may serve as imaging agents when labeled with (99m)Tc as shown for endostatin (Leung, 2004) and tumstatin (He et al., 2015) and for tumstatin conjugated to iron oxide nanoparticles (Ho et al., 2012).

## Author contributions

SV drafted the Section Receptors and Co-receptors of Matricryptins and Table 1 and made the figure. SB made bibliographical searches for all the sections and wrote the manuscript.

### Conflict of interest statement

The authors declare that the research was conducted in the absence of any commercial or financial relationships that could be construed as a potential conflict of interest.

## Abbreviations

ECM: extracellular matrix
EGF: epidermal growth factor
ERK: extracellular signal-regulated kinase
FAK: focal adhesion kinase
HA: hyaluronan
MAPK: mitogen-associated protein kinase
MMP: matrix metalloproteinase
mTOR: mammalian target of rapamycin
RHAMM: receptor for hyaluronic acid-mediated motility
TLR: toll-like receptor
VEGF: vascular endothelial growth factor
VEGFR: vascular endothelial growth factor receptor.
